# Incidence of Invasive and Noninvasive Pneumococcal Pneumonia Hospitalizations in People Aged ≥50 Years: Assessing Variability Across Denmark and Spain

**DOI:** 10.1093/infdis/jiae088

**Published:** 2024-03-09

**Authors:** Mónica López-Lacort, Marzyeh Amini, Hanne-Dorthe Emborg, Jens Nielsen, Scott A McDonald, Palle Valentiner-Branth, Javier Díez-Domingo, Alejandro Orrico-Sánchez

**Affiliations:** Vaccine Research Department of Fisabio-Public Health, Valencia, Spain; CIBER de Epidemiología y Salud Pública, Instituto de Salud Carlos III, Madrid, Spain; P95 Epidemiology and Pharmacovigilance, Leuven, Belgium; Department of Infectious Disease Epidemiology and Prevention, Statens Serum Institut, Copenhagen, Denmark; Department of Infectious Disease Epidemiology and Prevention, Statens Serum Institut, Copenhagen, Denmark; Centre for Infectious Disease Control, National Institute for Public Health and the Environment, Amsterdam, The Netherlands; Department of Infectious Disease Epidemiology and Prevention, Statens Serum Institut, Copenhagen, Denmark; Vaccine Research Department of Fisabio-Public Health, Valencia, Spain; CIBER de Epidemiología y Salud Pública, Instituto de Salud Carlos III, Madrid, Spain; Chair of Vaccines, Universidad Católica de Valencia San Vicente Mártir, Valencia, Spain; Vaccine Research Department of Fisabio-Public Health, Valencia, Spain; CIBER de Epidemiología y Salud Pública, Instituto de Salud Carlos III, Madrid, Spain; Chair of Vaccines, Universidad Católica de Valencia San Vicente Mártir, Valencia, Spain

**Keywords:** pneumonia, *Streptococcus pneumoniae*, pneumococcus, epidemiology, adults

## Abstract

Determining pneumococcal pneumonia (PP) burden in the elderly population is challenging due to limited data on invasive PP (IPP) and, in particular, noninvasive PP (NIPP) incidence. Using retrospective cohorts of adults aged ≥50 years in Denmark (2 782 303) and the Valencia region, Spain (2 283 344), we found higher IPP hospitalization rates in Denmark than Valencia (18.3 vs 9/100 000 person-years [PY], respectively). Conversely, NIPP hospitalization rates were higher in Valencia (48.2 vs 7.2/100 000 PY). IPP and NIPP rates increased with age and comorbidities in both regions, with variations by sex and case characteristics (eg, complications, mortality). The burden of PP in adults is substantial, yet its true magnitude remains elusive. Discrepancies in clinical practices impede international comparisons; for instance, Valencia employed a higher frequency of urinary antigen tests compared to Denmark. Additionally, coding practices and prehospital antibiotic utilization may further influence these variations. These findings could guide policymakers and enhance the understanding of international disparities in disease burden assessments.

## Teaser

The burden of pneumococcal pneumonia (PP) requiring hospitalization among older adults in Europe remains considerable. Estimates of invasive and noninvasive PP incidence among European countries vary between 2 and 6 times. Country-specific clinical practice hampers comparability of pneumonia in Europe.

Community-acquired pneumonia (CAP) is a significant cause of morbidity and mortality worldwide [1–3]. *Streptococcus pneumoniae* is the most common pathogen in CAP, accounting for one-third of cases [4, 5]. The burden of pneumococcal disease follows a U-shaped curve, with the greatest incidence and mortality in young children aged <5 years and older adults aged ≥65 years [6]. Pneumococcal pneumonia (PP) can be classified into invasive (IPP) and noninvasive (NIPP), depending on whether the infection is detected in normally sterile or nonsterile body sites [7].

The true burden of PP remains undetermined [8]. Some previous studies have reported the incidence of PP [9–11], mainly relying on IPP because it is a reportable condition in many countries [12]. However, there are important data gaps pertaining to NIPP [13], as most PP cases are not associated with detected bacteremia [14]. Relying on IPP data alone could potentially underestimate the overall burden of PP, as it has been suggested that for every case of adult IPP, there are at least 3 additional cases of NIPP [5].

On the other hand, the type of testing employed for pneumococcal detection has been shown to play an important role, where traditional detection methods such as blood and/or sputum culture alone are likely to underestimate the burden of pneumococcus [3]. Testing practices, which may differ between countries, are intrinsically correlated with a diagnosis of IPP and NIPP. Other healthcare practices (eg, hospital admission criteria, disease management, and coding practices) may also depend on regional health system organization and by their social and economic circumstances, directly impacting the population's health and needs for healthcare [15]. Therefore, international comparisons may account for some of these contextual differences, which may impact the incidence of PP [16]. Multicountry studies are needed to identify potential disparities in the epidemiology of PP that allow estimating the real burden of IPP and NIPP. These data will enable forecasting, public health decision-making, and allocation of resources, which are essential for developing effective vaccination strategies in adults.

In this context, the Valencia region of Spain and Denmark—2 regions with substantial expertise in retrospective disease burden studies—are participating in the second Innovative Medicine Initiative (IMI2)–supported project called “Vaccines and InfecTious Diseases in the Ageing PopuLation” (VITAL) [16], which aims to address the current challenges of (potentially) vaccine-preventable diseases in the ageing European population.

This study aims to assess the variability in the incidence of IPP and NIPP in adults aged ≥50 years in Denmark and the Valencia region of Spain. This article also discusses the complexity of comparing epidemiological health data obtained with data sources from different regions or countries.

## METHODS

### Study Design, Population, and Setting

This is a population-based, retrospective dynamic cohort study using electronic healthcare registries. All adults aged ≥50 years covered by the public health systems and residing in the Valencia region of Spain (hereafter “Valencia”) and in Denmark from 1 January 2010 up to 31 December 2018 were included. The 2 regions have a similar population size of approximately 5 million inhabitants, with about 50% being >50 years of age in each region. Overall, there are 40 and 24 public hospitals in Denmark and Valencia, respectively, with a rate of available beds per capita of 2.5 and 2.1 per 1.000 inhabitants, respectively [17, 18]. The majority of healthcare services are financed by general taxes and mainly provided free of charge in both regions.

During the study period, the 23-valent pneumococcal polysaccharide vaccine was publicly funded for immunocompetent individuals ≥65 years old at risk of pneumococcal disease [19], 13-valent pneumococcal conjugate vaccine (PCV13) was publicly funded for high-risk individuals [20], and infants had universal free 7-valent PCV/PCV13 or PCV13 publicly funded during the study period [21]. Vaccine coverage rates were <20% in adults and >90% in children in Valencia [22] and Denmark [23].

### Follow-up

The study populations were followed up since the first date of registration in the database (The Valencian population information system or the Danish vivil registration system) or date of the 50th birthday (whichever occurred later). End of follow-up was the date of death, disenrollment from registries (ie, moving), or end of the inclusion period (31 December 2018), whichever occurred first. As Valencia is a coastal region with many tourists, subjects with <6 months’ registration in the databases were excluded from the study.

### Data Sources

Electronic healthcare registries from Valencia (Valencia Health System Integrated Databases [VID]; Supplementary Figures S1 and S2) [24] and the database from the Statens Serum Institut in Copenhagen, Denmark, were used. Details of databases are described in the Supplementary Materials. Of note, Valencia included medical information for each patient attended in the primary care setting (general practitioners and specialists) for detection of underlying conditions; no primary care data were available for Denmark. Information from all registries was linked at the individual level through a unique personal identification number in each country.

### Case Definitions

Incident IPP cases were defined as the first hospitalization per person with a diagnosis of pneumonia or pneumonia-related complications (Supplementary Table 1) in any diagnosis position with a laboratory-confirmed pneumococcus result isolated from blood or another normally sterile site at admission date (±30 days). Normally sterile sites include blood, cerebrospinal fluid, pleural fluid, peritoneal fluid, pericardial fluid, bone, synovial fluid, and internal body sites.

Incident NIPP cases were defined as the first hospitalization per person with a discharge diagnosis of pneumonia or pneumonia-related complications (Supplementary Table 1) in any diagnosis position, with a positive pneumococcal urinary antigen test and negative or missing laboratory confirmation for pneumococcus/other bacteria from normally sterile sites ±30 days from diagnosis day.

Incident PP cases were defined as the first hospitalization for IPP or NIPP per person during the study period.

### Variables of Interest

The following variables were studied: sex, calendar year, age group (50–54, 55–59, 60–64, 65–69, 70–74, 75–79, ≥80) and nationality (Danish, Spanish, or other), as well as underlying conditions (diabetes mellitus, cardiovascular disease, and chronic lung disease [CLD]) and complications (sepsis, cerebrovascular events, cardiovascular events, acute kidney injury, and respiratory failure/acute respiratory distress syndrome [ARDS]). The *International Classification of Diseases* (*ICD*) Ninth or Tenth Revision diagnosis codes of the underlying conditions and complications are provided in Supplementary Tables 2 and 3. In Valencia, underlying conditions were identified by systematic search of *ICD* codes from hospital or ambulatory databases during the study period. In Denmark, underlying conditions could be identified in the hospital and emergency rooms datasets only, where the 5 years prior to the beginning of the study period were also searched. The identification of acute complications (parapneumonic effusion, empyema, bronchopleural fistula, lung abscess, necrotizing pneumonia, and pulmonary gangrene) and other complications (sepsis, cerebrovascular or cardiovascular events, acute kidney disease, or ARDS) within 180 days was restricted to hospital databases in both countries. PP-associated deaths were also studied and defined as those occurring during the hospitalization or within 30 days since the PP diagnosis.

### Statistical Analyses

Characteristics of the populations (sex, age, nationality, mortality, and underlying conditions) were described by frequencies and proportions. Incidence rates (IRs) of hospitalizations for PP, IPP, and NIPP were estimated by 5-year age groups, sex, underlying conditions, and calendar year. IRs were calculated as the number of incident IPP, NIPP, or PP cases divided by the person-years (PY) at risk. IRs were expressed as cases per 100 000 PY, and exact Poisson 95% confidence intervals (CIs) were estimated. The characteristics of the IPP and NIPP incident cases regarding the age, sex, length of hospital and intensive care unit (ICU) stay (only Valencia), mortality, complications, sequelae, and comorbidities were also presented. Categorical variables were described by frequencies and proportions, and continuous variables by mean with standard deviation or median with interquartile range.

All analyses were done using R version 4.2.0 (for Valencia) or SAS 9.4 (SAS Institute, Cary, North Carolina) (for Denmark) software.

### Ethical Statement

Each local study was approved by national, regional, or institutional ethics committees, as appropriate. The protocol of this study was approved by the ethics committee of the Arnau de Vilanova Hospital of Valencia. According to Danish law, ethical approval or individual consent is not required for anonymized aggregated register-based studies. This observational study used retrospective anonymized nonidentifiable data transferred from the Valencian and Danish ministries of health to the research teams, according to Spanish and Danish laws and institutional requirements. According to the law, this kind of registry-based research is exempt from obtaining patient informed consent; both ethics committees accepted this exemption.

## RESULTS

### Characteristics of the Study Population

Demographic characteristics of the studied population by study site are shown in Table 1. Overall, 2 283 344 and 2 782 303 persons met the inclusion criteria in Valencia and Denmark during the study period, respectively. This contributed a total of 15 502 325 and 19 287 085 PY in Valencia and Denmark, respectively. The distribution of population by both sex and age groups were similar between countries. In Valencia, the proportion of people with underlying conditions (65%) was substantially higher than in Denmark (35%). The proportion of population vaccinated against with any pneumococcal vaccine was 4% and 6% in Valencia and Denmark, respectively.

**Table 1.** Characteristics of Study Populations in Valencia Region, Spain and Denmark

Characteristic	Valencia, Spain (N = 2 283 344)	Denmark (N = 2 782 303)
Sex		
Male	1 073 835 (47)	1 349 147 (48.5)
Female	1 209 509 (53)	1 433 156 (51.5)
Age at entry, y		
50–54	971 370 (43)	1 115 376 (40)
55–59	274 169 (12)	359 072 (13)
60–64	265 037 (12)	380 576 (14)
65–69	231 694 (10)	306 846 (11)
70–74	177 734 (8)	222 755 (8)
75–79	161 179 (7)	164 428 (6)
≥80	202 161 (9)	233 250 (8)
Nationality		
Other	217 673 (10)	225 804 (8)
Spaniard/Danish	2 063 895 (90)	2 556 280 (92)
Unknown	1776 (0.07)	219 (0.01)
Died during the study period	338 341 (15)	475 565 (17)
Underlying condition^a^		
Diabetes	491 976 (22)	226 297 (8)
CVD	1 268 979 (56)	858 719 (31)
CLD	501 450 (22)	152 958 (5)
Any^b^	1 478 007 (65)	986 117 (35)
Pneumococcal vaccination^c^	93 462 (4.1)	162 062 (5.8)
Follow-up		
Median of follow-up (IQR)	9.00 (4.53–9.00)	9.00 (4.83–9.00)
Sum of total follow-up time	15 502 325	19 287 085

Data are presented as No. (%) unless otherwise indicated.

Abbreviations: CLD, chronic lung disease; CVD, cardiovascular disease; IQR, interquartile range.

^a^Underlying conditions during follow-up.

^b^Any includes any of the comorbidities studied (diabetes, CVD, or CLD).

^c^Proportion of individuals people aged ≥50 years vaccinated in the cohort with any pneumococcal vaccine at any time during the study period.

### Incidence Rate of IPP Hospitalizations

A total of 1398 IPP incident cases in Valencia and 3536 in Denmark were identified. Table 2 shows that the overall IR of IPP in Denmark was higher than in Valencia (18.3 vs 9 per 100 000 PY). In Valencia, the IR in men (12.1) was almost 2 times higher than in women (6.4). In Denmark, the rate was similar between sexes. Incidence increased by age and underlying condition in both regions, being at least 7 times higher in people ≥80 years than in those aged 50–55 years. Incidence in those with chronic underlying conditions was generally higher than the overall population, and patients with CLD had the highest IR. Overall, the ratio of IPP disease in Denmark and Valencia ranged between 1.7 and 2.8 for the different age groups, as well as for those with diabetes and cardiovascular disease. The IR for those with CLD was >4 times higher in Denmark than Valencia. Fluctuations in IPP IRs were noticed over calendar time in both countries, showing a decreasing IR over time in both regions, while Valencia showed a recent increase in incidence in 2018. IRs by age, sex, comorbidities, and calendar year are shown in Table 2.

**Table 2.** Hospitalized Invasive Pneumococcal Pneumonia Incidence Rates in Valencia, Spain and Danish Study Populations, by Site, Sex, Age Group, Underlying Conditions, and Year

Characteristic	Valencia, Spain			Denmark		
	No. of (First) Cases	Follow-up Period	Incidence Rate (95% CI) per 100 000 PY	No. of (First) Cases	Follow-up Period	Incidence Rate (95% CI) per 100 000 PY
Overall	1398	15 499 174	9 (8.55–9.51)	3536	19 276 707	18.3 (17.7–19.0)
Sex						
Male	866	7 132 580	12.1 (11.35–12.98)	1749	9 225 526	19.0 (18.1–19.9)
Female	532	8 366 593	6.4 (5.83–6.92)	1787	10 051 181	17.8 (17.0–18.6)
Age at entry, y						
50–54	107	3 089 660	3.5 (2.84–4.18)	209	3 538 619	5.9 (5.1–6.8)
55–59	125	2 680 749	4.7 (3.88–5.56)	306	3 227 527	9.5 (8.4–10.6)
60–64	161	2 354 755	6.8 (5.82–7.98)	413	3 118 451	13.2 (12.0–14.6)
65–69	179	2 139 087	8.4 (7.19–9.69)	517	3 053 515	16.9 (15.5–18.5)
70–74	187	1 774 884	10.5 (9.08–12.16)	590	2 452 639	24.1 (22.2–26.1)
75–79	212	1 439 089	14.7 (12.82–16.85)	463	1 688 974	27.4 (25.0–30.0)
≥80	427	2 020 950	21.1 (19.17–23.23)	1038	2 196 982	47.2 (44.4–50.2)
Underlying condition						
Diabetes	504	2 842 573	17.7 (16.22–19.35)	390	993 935	39.2 (35.4–43.3)
CVD	1007	7 758 785	13.0 (12.19–13.81)	1315	3 587 050	36.7 (34.7–38.7)
CLD	583	2 368 815	24.6 (22.65–26.69)	518	498 719	103.9 (95.1–113.23)
Year						
2010	176	1 573 924	11.2 (9.59–12.96)	466	2 022 615	23.0 (21.0–25.2)
2011	167	1 608 511	10.4 (8.87–12.08)	447	2 046 560	21.8 (19.9–24.0)
2012	142	1 647 408	8.6 (7.26–10.16)	449	2 078 572	21.6 (19.6–23.7)
2013	136	1 679 986	8.1 (6.79–9.58)	344	2 100 620	16.4 (14.7–18.2)
2014	137	1 720 644	8 (6.68–9.41)	345	2 135 037	16.2 (14.5–18.0)
2015	132	1 760 346	7.5 (6.27–8.89)	379	2 169 727	17.5 (15.8–19.3)
2016	135	1 804 778	7.5 (6.27–8.85)	369	2 212 901	16.7 (15.0–18.5)
2017	161	1 832 646	8.8 (7.48–10.25)	375	2 242 008	16.7 (15.1–18.5)
2018	212	1 870 933	11.3 (9.86–12.96)	362	2 268 668	16.0 (14.3–17.7)

Abbreviations: CI, confidence interval; CLD, chronic lung disease; CVD, cardiovascular disease; PY, person-years.

### Incidence Rate of NIPP Hospitalizations

A total of 7467 NIPP incident cases in Valencia and 1391 cases in Denmark were identified. Table 3 shows that the overall IR of IPP in Valencia was 7 times higher than in Denmark (48.2 vs 7.2 per 100 000 PY). Differences in IR by sex were found in Valencia but not in Denmark. IR increased by age, being between 8 and 10 times higher in the population aged ≥80 years than those aged 50–55 years. Overall, the ratio of NIPP disease in Denmark and Valencia ranged between 1.6 and 2.3 for the different age groups. IRs in the population with chronic underlying conditions were generally higher than the overall population, and patients with CLD had the highest IR. The ratio of IR for those with CLD was >4 times higher in Valencia than Denmark. Fluctuations in NIPP IRs were noticed over calendar time in both countries, showing an increasing IR over time in both regions. IRs by age, sex, comorbidities, and calendar year are shown in Table 3.

**Table 3.** Hospitalized Noninvasive Pneumococcal Pneumonia Incidence Rates in Valencia, Spain and Danish Study Populations, by Site, Sex, Age Group, Underlying Conditions, and Year

Characteristic	Valencia, Spain			Denmark		
	No. of (First) Cases	Follow-up Period	Incidence Rate (95% CI) per 100 000 PY	No. of (First) Cases	Follow-up Period	Incidence Rate (95% CI) per 100 000 PY
Overall	7467	15 487 451	48.2 (47.13–49.32)	1391	19 283 594	7.2 (6.8–7.6)
Sex						
Male	4135	7 126 500	58 (56.27–59.82)	666	9 228 723	7.2 (6.7–7.8)
Female	3332	8 360 950	39.9 (38.51–41.23)	725	10 054 871	7.2 (6.7–7.8)
Age at entry, y						
50–54	317	3 089 373	10.3 (9.16–11.46)	97	3 538 800	2.7 (2.2–3.3)
55–59	435	2 680 067	16.2 (14.74–17.83)	157	3 227 990	4.9 (4.1–5.7)
60–64	499	2 353 777	21.2 (19.38–23.14)	185	3 119 306	5.9 (5.1–6.8)
65–69	724	2 137 852	33.9 (31.44–36.43)	193	3 054 719	6.3 (5.5–7.3)
70–74	925	1 773 223	52.2 (48.86–55.64)	215	2 453 938	8.8 (7.6–10.0)
75–79	1071	1 437 200	74.5 (70.12–79.12)	186	1 689 879	11.0 (9.5–12.7)
≥80	3496	2 015 959	173.4 (167.71–179.26)	358	2 198 961	16.3 (14.6–18.1)
Underlying condition						
Diabetes	2621	2 838 170	92.3 (88.85–95.95)	164	994 948	16.5 (14.1–19.2)
CVD	5714	7 749 240	73.7 (71.84–75.67)	465	3 590 056	13.0 (11.8–14.2)
CLD	3637	2 361 634	154 (149.04–159.09)	226	499 898	45.2 (39.5–51.5)
Year						
2010	626	1 573 725	39.8 (36.72–43.02)	119	2 022 757	5.9 (4.9–7.0)
2011	756	1 607 973	47 (43.72–50.49)	132	2 046 967	6.4 (5.4–7.6)
2012	665	1 646 576	40.4 (37.38–43.58)	92	2 079 210	4.4 (3.6–5.4)
2013	736	1 678 926	43.8 (40.73–47.12)	179	2 101 410	8.5 (7.3–9.9)
2014	817	1 719 277	47.5 (44.32–50.89)	173	2 135 869	8.1 (6.9–9.4)
2015	895	1 758 751	50.9 (47.61–54.33)	184	2 170 632	8.5 (7.3–9.8)
2016	835	1 802 979	46.3 (43.22–49.56)	165	2 213 881	7.5 (6.4–8.7)
2017	957	1 830 669	52.3 (49.02–55.7)	143	2 243 067	6.4 (5.4–7.5)
2018	1180	1 868 576	63.1 (59.6–66.86)	204	2 269 800	9.0 (7.8–10.3)

Abbreviations: CI, confidence interval; CLD, chronic lung disease; CVD, cardiovascular disease; PY, person-years.

### Incidence Rate of PP Hospitalizations

A total of 8865 and 4927 IPP or NIPP incident cases were identified in Valencia and Denmark, respectively (Table 4). Overall, the IR of PP was approximately 2 times higher in Valencia than in Denmark (57 vs 25.4/100 000 PY). That incidence rises to 37.6 and 97.6 per 100 000 PY in the population >65 years of age in Denmark and Valencia, respectively (Supplementary Table 4).

**Table 4.** Characteristics of Invasive and Noninvasive Pneumococcal Pneumonia Incident Cases in Valencia, Spain and Danish Study Populations

Characteristic	Valencia, Spain		Denmark	
	IPP (n = 1398)	NIPP (n = 7467)	IPP (n = 3536)	NIPP (n = 1391)
Age, y, median (IQR)	73 (63–81)	78 (69–85)	72 (64–81)	71 (62–80)
Female sex	532 (38)	3852 (52)	1787 (51)	725 (52)
LOS, d, median (IQR)	9 (6–16)	7 (4–12)	5.4 (1.71–11.4)	5 (2–12)
ICU stay	236 (17)	626 (8.4)	NA	NA
ICU LOS, d, median (IQR)	1.00 (1.00–3.00)	1.00 (1.00–3.00)	NA	NA
Mortality within 30 d	265 (19)	1325 (18)	405 (11)	168 (12)
Acute complications^a^	291 (20.8)	721 (9.7)	387(11)	98 (7)
Other complications	1073 (77)	5499 (74)	371 (10)	179 (13)
Sepsis	252 (18)	468 (6.3)	68 (2)	23 (2)
Cerebrovascular events	73 (5.2)	519 (7.0)	43 (1)	28 (2)
Cardiovascular events	531 (38)	2923 (39)	244 (7)	88 (7)
Acute kidney failure	254 (18)	1159 (16)	12 (0.3)	80 (0.6)
Respiratory failure/ARDS	552 (39)	3144 (42)	77 (2)	58 (4)
Comorbidities				
DM	531 (38)	2748 (37)	390 (11)	164 (12)
CVD	1056 (76)	5930 (79)	1315 (37)	465 (33)
CLD	644 (46)	3907 (52)	518 (15)	226 (16)

Data are presented as No. (%) unless otherwise indicated. Details of *International Classification of Diseases Codes (ICD)* used for complications and comorbidities are shown in the Supplementary Material.

Abbreviations: ARDS, acute respiratory distress syndrome; CLD, chronic lung disease; CVD, cardiovascular disease; DM, diabetes mellitus; ICU, intensive care unit; IPP, invasive pneumococcal pneumonia; IQR, interquartile range; LOS, length of stay; NA, not available; NIPP, noninvasive pneumococcal pneumonia.

^a^Acute complications (parapneumonic effusion, empyema, bronchopleural fistula, necrotizing pneumonia, lung abscess, and pulmonary gangrene).

### Characteristics of the IPP and NIPP Hospitalizations

Table 4 shows the characteristics of the IPP and NIPP incident hospital cases. The median age of cases of IPP and NIPP hospitalizations was 72 and 71 years for Denmark and 73 and 78 years for Valencia. Median lengths of IPP and NIPP hospital stays were higher in Valencia (9 and 7 days, respectively) than in Denmark (5.4 and 5 days, respectively). In Valencia, 8% and 17% of NIPP and IPP cases, respectively, required a stay in the ICU. Mortality within 30 days after either IPP or NIPP was higher in Valencia (around 18%) than Denmark (around 11%). In Denmark, 11% and 7% of the IPP and NIPP respective cases presented with any acute complication, whereas these percentages were lower in Spain (8% and 6%, respectively). Furthermore, 10%–13% of the Danish cases had at least 1 of the studied other complications; however, >70% of the cases in Spain presented at least 1 hospitalization of the studied other complications. The most prevalent complication was cardiovascular events, followed by respiratory failure/ARDS.

### Testing Procedures in 2 Regions

As observed in Table 5, the total number of antigen pneumococcal tests requested in Valencia was between 2.5 and 4 times higher than Denmark. The proportion of positive antigen tests was between 1.5 and 2.5 times higher in Valencia than Denmark. However, the yearly number of positive culture tests in Denmark was around 4 times higher than in Valencia. Overall, the ratio of positive culture/antigen in Valencia was approximately 1.0 and in Denmark ranged from approximately 13 to 40. Among positive IPP cases, 84% and 98% were from blood specimens in Valencia and Denmark, respectively.

**Table 5.** Number of Laboratory Results for Pneumococcal Pneumonia Diagnosis by Type of Test, Site, and Year

Year	Valencia, Spain					Denmark				
	Total Antigen	Positive Antigen	Antigen Positivity, %	Positive Culture	Ratio Culture/Antigen	Total Antigen	Positive Antigen	Antigen Positivity, %	Positive Culture	Ratio Culture/Antigen
2010	20 221	1657	8.2	1900	1.15	4933	256	5.2	9862	38.5
2011	23 427	1838	7.8	2010	1.09	6704	321	4.8	8685	27.1
2012	2 319	1478	6.4	1822	1.23	6067	215	3.5	8526	39.7
2013	21 478	1689	7.9	1805	1.07	7008	444	6.3	8204	18.5
2014	23 095	1882	8.1	1817	0.97	9502	427	4.5	7587	17.8
2015	26 293	2179	8.3	1925	0.88	10 117	410	4.1	7748	18.9
2016	27 338	2101	7.7	1847	0.88	10 428	387	3.7	7227	18.7
2017	30 646	2352	7.7	2119	0.90	11 739	345	2.9	5695	16.5
2018	31 892	2958	9.3	2553	0.86	13 077	469	3.6	5994	12.8

Antigen refers to urinary antigen tests. Positive culture refers to bacterial growth in cultures.

## DISCUSSION

It is difficult to determine the burden of PP because scant papers have published data on the incidence of (N)IPP specifically, rather than overall pneumococcal disease. In this large population-based cohort study, we assessed the variability in incidence of IPP and NIPP hospitalizations in the aging population across 2 European regions. Large differences in (N)IPP incidence between regions were found; while IPP IR was higher in Denmark than Valencia (18.3 vs 9 per 100 000 PY), NIPP IR was the opposite (7 vs 48 per 100 000 PY, respectively). Such epidemiological differences may be related to variations in clinical practice and management of pneumococcal disease in each region, which hampers international comparisons.

IPP estimates in Valencia align with the national reports for notifiable diseases, supporting the reliability of the data, while only data on invasive pneumococcal disease are available for Denmark [19, 25]. Despite the variability, the incidence of IPP increased by age and underlying conditions in both regions. A rise in incidence and hospitalization for pneumonia, especially in the elderly population, has been studied in some European countries [26–28]. These studies show a high prevalence of comorbidities in elderly populations, which have been linked to a poorer prognosis for pneumonia [29, 30]. Large sex differences in the (N)IPP IR were found in Valencia but not in Denmark. Previous studies already confirmed the importance of sex as an epidemiological factor in PP and pneumococcal disease [9, 31]. However, other previous Danish studies for invasive pneumococcal disease did not find such a difference [32]. Other factors such as differences in immunity [33–35], comorbid conditions [1, 2], or the use of tobacco [36] may have played a role in those sex differences.

The overall IR of PP hospitalizations was 25 and 57 per 100 000 PY in Denmark and Valencia, respectively. Comparable incidence of PP was estimated in adults aged ≥65 years in Sweden (20–60 cases/100 000 PY) [37] and Italy (13.4–113.3/100 000 PY) [38]. However, 2 studies in the Catalonia region of Spain have estimated a higher PP IR (82–90 per 100 000) in the same age group [9, 10]. Differences in the case definition (eg,. inclusion of sputum cultures) or the period studied (2015 and 2017–2018, respectively) could have influenced the incidence. Finally, much higher IRs have been published in the United States [11]. Overall, important differences were found across countries in PP IRs (ranging from 68 to 7000 cases/100 000 PY), in both our study and others’ [2]. These variability could be in part due to difficulties in characterizing nonbacteremic PP cases [5].

Depending on the region, between 15% and 70% of the overall PP cases were classified as NIPP in Denmark and Valencia, respectively. In Valencia, the ratio of overall hospitalizations for NIPP/IPP was 5.3, while in Denmark it was 0.4. It has been previously estimated that >70% of cases of PP are noninvasive [5, 39]. Another recent study in Spain also found that the incidence of NIPP was 7 times higher than IPP [10]. Different factors such as clinical practice, pathways of care, access to resources, prevalence of comorbidities, codification, and registration of diseases may influence the IR. Further challenges arise from reliable diagnostic testing of pneumonia due to the lack of a universally recognized case definition [39]. For instance, traditional detection methods such as blood and/or sputum culture are likely to underestimate burden, while studies that are heavily reliant on urinary antigen tests may overestimates cases [3]. Here, we show that the use of urinary antigen tests in Valencia was between 2.5 and 4 times higher than Denmark, and the opposite occurred with culture tests. In addition, the administration of prediagnostic use of antibiotics can also impair the comparability between the 2 countries [26, 40, 41]. Urinary antigen tests are not affected by prediagnostic use of antibiotics [42]. The European surveillance report of antimicrobial consumption showed that Spain consumes 40% more antibiotics than Denmark (20 vs 13 defined daily dose per 1000 inhabitants in 2021) [43]. In the same line, the 2010 guidelines for antibiotic use in Spain did not recommend an etiological study for patients with nonsevere CAP receiving outpatient antibiotic treatment [44]. These reasons might explain, at least in part, the differences in (N)IPP IRs between regions. The low pneumococcal vaccination coverage in both regions during the study period must not have had a major impact on these differences.

Prevalence of comorbid conditions in the 2 cohorts may have also influenced the estimation of (N)IPP IRs and, potentially, the almost double mortality rates found in Valencia. This prevalence was substantially higher in the overall population of Valencia (22%–65%) than Denmark (8%–36%) (Table 1). But the main cause for such differences must be the sources of information used, where Valencia, unlike Denmark, also used primary databases in addition to hospital databases for the determination of comorbidities. Nevertheless, when comorbidities are studied only in the hospital registries in both regions, there is still a 60% higher prevalence in Valencia than Denmark (Table 4). This highlights that other differences, such as coding, could have also played a major role; although *ICD* is an international standard for clinical purposes, using *ICD* codes has a limited ability to compare across countries since most use their own adapted version [45]. Moreover, variable mortality rates among countries may be linked to potential selection bias in Spain, where frequent antibiotic administration by general practitioners may imply that admitted patients are the most severe, leading to higher mortality and complications. This article presents important lessons learned when comparing epidemiological data between regions or countries. Here, we showed that, despite working under the same protocol and case definitions, we have identified other key factors to consider when studying disease burden. Differences in clinical practice such as testing practice, antibiotic use, admission rates, and coding between regions may be key elements when comparing results from different datasets. The use of different sources of information (eg, primary care) must also be considered to avoid an underestimation of comorbidities. Nevertheless, health systems operate in their own national/regional context. Therefore, other potential explanations for differences between the 2 countries in our study may include exposure to social, economic, or environmental risk factors, differences in standards of care, thresholds for hospital admission, clinical judgment, or even artifactual [46].

The main strength of the study is the use of high-quality population-based registries at the individual level in 2 regions with substantial experience in retrospective epidemiological studies with real-world data. However, this study also presents several limitations; databases are subject to the limitations inherent to routine clinical practice and electronic reporting. Here, there may be information bias due to incomplete data or differences in data recording practices (eg, data accuracy, misclassification, heterogeneity). However, this is an intrinsic problem of any repository using data from routine clinical practice. Potential limitations of the VID have been described elsewhere [24]. Also, databases may not always contain information on people who are not in contact with the public healthcare service or who are privately attended. In Valencia, only the public sector population is recorded, whereas Denmark included both public and private hospitals.

## CONCLUSIONS

During 2010–2018, the burden of PP requiring hospitalization among older adults in Europe remained considerable. The true IR of PP is still unknown, while wide variations in the incidence of IPP and NIPP are found between European countries because estimations are a mere reflection of their clinical practice (eg, antibiotic use, microbiological testing, coding practices, and admission criteria). Those differences could have led to an underestimation of the burden of IPP and NIPP in Valencia and Denmark, respectively. Acknowledging and addressing these challenges is crucial for meaningful cross-country assessments and the development of effective vaccination strategies.

## Supplementary Data

Supplementary materials are available at *The Journal of Infectious Diseases* online (http://jid.oxfordjournals.org/). Supplementary materials consist of data provided by the author that are published to benefit the reader. The posted materials are not copyedited. The contents of all supplementary data are the sole responsibility of the authors. Questions or messages regarding errors should be addressed to the author.

## Supplementary Material

jiae088_Supplementary_Data

## Notes

***Acknowledgments.*** The authors acknowledge Alexandria Williams (P95) for manuscript editing support; Dr Francisco Sanz (medical director at Hospital General Universitario de Valencia, Spain) for his contribution to results interpretation; members of Work Package 1 (Anneke Steens, Kelly Jhonson, and Christian Theilacker) for their contributions; and Comisión PROSIGA, Conselleria de Sanitat, Generalitat Valenciana for data extraction.

***Author contributions*.** A. O.-S. is the guarantor of the paper, taking responsibility for the integrity of the work as a whole, from inception to published article. M. L.-L. contributed to data acquisition, cleaning, analysis, and interpretation; drafting the article and revising it critically for important intellectual content; and final approval of the version to be published. M. A. contributed to data interpretation; drafting the article and revising it critically for important intellectual content; and final approval of the version to be published. J. D.-D. contributed to study conception and design; data acquisition, analysis, and interpretation; drafting the article; and final approval of the version to be published. H.-D. E., J. N., and P. V.-B. contributed to data acquisition, cleaning, analysis, and interpretation; revising the article critically for important intellectual content; and final approval of the version to be published. S.A.D. contributed to study conception and design, and interpretation; and final approval. All authors have read and approved the manuscript.

***Financial support*.** The VITAL project has received funding from the Innovative Medicines Initiatives 2 Joint Undertaking (grant agreement number 806776) and the Dutch Ministry of Health, Welfare and Sport. The Joint Undertaking receives support from the European Union’s Horizon 2020 research innovation program and EFPIA members.
