# Supplementary material for: Incidence of Invasive and Noninvasive Pneumococcal Pneumonia Hospitalizations in People Aged ≥50 Years: Assessing Variability Across Denmark and Spain
Source: J Infect Dis. 2024 Mar 9;230(3):e559–67. doi: 10.1093/infdis/jiae088 (PMC11420809; doi:10.1093/infdis/jiae088)
Supplement: jiae088_Supplementary_Data [file jiae088_supplementary_data.docx]

Supplementary data

Electronic Health Records (EHR), Databases

The Valencia Integrated Databases (VID)

VID is a set of multiple, public, population-wide electronic databases for the Valencia Region, the fourth most populated Spanish region, with approximately 5 million inhabitants and an annual birth cohort of 48,000 new-borns, representing 10.7% of the Spanish population and around 1% of the European population. The VID provides exhaustive longitudinal information including sociodemographic data, clinical, pharmaceutical (prescription, dispensation) and healthcare utilization data from hospital care, emergency departments, specialised care, primary care and other public health services. All the information in the VID datasets can be linked at the individual level through a single personal identification code.


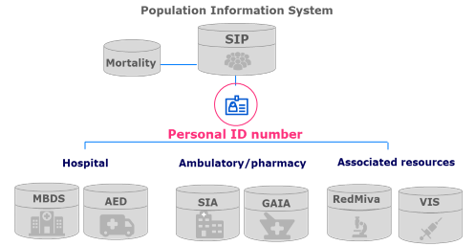


Figure S1. The Valencia Health System Integrated Database (VID); VIS, Vaccine Information System; RedMIVA, Microbiological Surveillance System; MBDS, Minimum Basic hospital Data Set; AED, Accident & Emergency Department record; GAIA, Pharmaceutical Module; SIA, Ambulatory Information System.

Useful databases for the study are described below:

Population Information System (SIP)

SIP is a region-wide database that provides basic information on Valencia Health System (VHS) coverage (insurance modality, pharmaceutical copayment status, assigned Healthcare Department, Primary Healthcare District, and primary care doctor, etc.) and also some sociodemographic (sex, date of birth, nationality, country of origin, risk of social exclusion, geographic location, address, etc). The SIP database is paramount to the VID as it is the source of the individual, exclusive and permanent identifier number associated to each individual (the SIP number) that is then used throughout the rest of the databases, allowing data linkage across the multiple databases in the network.

Ambulatory Medical Record (ABUCASIS)

ABUCASIS was implemented in 2006 for primary and specialized outpatient activity, reaching 96% population coverage from 2009. ABUCASIS is integrated by two main modules: the Ambulatory Information System (SIA) and the Pharmaceutical Module (GAIA). This database contains primary care diagnoses and procedures (physician coded using the International Classification of Diseases 9th Revision, Clinical Modification (ICD-9-CM)) and all drug prescriptions (using Anatomical Therapeutic Chemical (ATC) Classification System).

Hospital Medical Record

The Hospital Medical Record includes the Minimum Basic Data Set at Hospital Discharge (MBDS) and the Accident & Emergency Department (AED) clinical record. The MBDS is a synopsis of clinical and administrative information on all hospital admissions and major ambulatory surgery in the Valencia Health System (VHS) hospitals, including public-private partnership hospitals (around 450,000 admissions per year in the region). The MBDS includes admission and discharge dates, age, sex, geographical area and zone of residence, main diagnosis at discharge, up to 30 secondary diagnoses (comorbidities or complications) and clinical procedures performed during the hospital episode. It also includes the Diagnosis Related Groups (DRG; a system to classify hospital cases into one of approximately 500 groups, expected to have similar hospital resource use) assigned at discharge. Data are considered reliable since 2002. The Accident & Emergency Department clinical record was launched in 2008 and collects triage data, diagnoses, tests and procedures performed in public emergency rooms. As with the MBDS, the coding system used was ICD9CM until December 2015 and ICD10ES afterwards. Diagnosis codification has been increasing from about 45% of all AED visits between 2008 and 2014 up to around 75% in 2017, basically due to the progressive incorporation of hospital coding.

Microbiological Surveillance Network (RedMIVA)

RedMiva contains the results of the microbiological analyses performed in VHS. Data is transferred from the laboratories to the RedMIVA database on a daily basis, providing realtime detection of circulating microorganisms and resistance patterns, and enabling microbiological surveillance. Importantly, RedMIVA gathers not only positive but also negative determinations. This database has been available since 2008.

Vaccine Information System (SIV)

SIV stores all the information on vaccination in the VHS since 2000, though data are only considered reliable after 2005. Available data include vaccine by type, manufacturer, batch number, number of doses, location and administration date, adverse reactions related to vaccines, rejected vaccinations and, if applicable, risk groups.

In all databases in the VID, data are collected daily as a part of the routine clinical care provided to patients. Accordingly, data may be available for research until the data are extracted. Only in some cases, such as the MBDS and the AED records, are data subject to a consolidation and quality check process before data is available for research, so data from the last quarter before the data extraction may be missing or non-consolidated.

Database from the State Serum Institute (SSI)

Danish Civil Registration System (CRS)

The CRS database was initiated in 1968 and comprises individual level information on birth date, gender, vital status and sequential dates of migration of all residents in Denmark, i.e. approx. 5.6 mill. persons. Using a unique personal identifier assigned to all Danish residents upon birth or immigration makes it possible to link the CRS with health care databases including those described below. Update interval: daily.

The Danish National Patient Registry (DNPR)

The DNPR has recorded every inpatient hospitalization since 1977, and every outpatient and emergency room visit since 1995. For each admission, one primary and potentially several secondary diagnoses are registered according to the International Classification of Diseases, Eighth Revision (ICD-8) until the end of 1993, and Tenth Revision (ICD-10) thereafter. In addition, DNPR includes procedure codes. After admission to a hospital all movements between different departments are registered as well as length of stay at each department. Update intervals: daily, however as this is a discharge database, the records are available at discharge and not at admission.

The Danish Microbiology Database (MiBa)

The MiBa was established in 2010. This national database receives all microbiological test results from the clinical microbiological departments. All microbiological tests ordered by the GP’s and hospital physicians are analysed at the clinical microbiological departments and a copy of the test result is transferred to MiBa. This database includes information on unique identifier, date of sampling, identification of the clinical microbiological department who analysed the sample, specimen, the analyses performed and the test result. This database includes both positive and negative test results. Update intervals: several times a day.

The Danish Vaccination Registry (DVR)

The DVR contains individual level data on vaccines given as part of the national childhood vaccination programme, the influenza vaccination programme and the HPV vaccination programme. Until November 2015, information on all vaccines bought at the pharmacy on prescription were imported into the DVR. From November 2015, GPs and others authorized to vaccinate have an obligation to record all vaccinations in DVR. DVR contains information on Date of vaccination, type of vaccine, unique personal identifier, identification of vaccinator, product name, ATC code, dose and batch number. Update intervals: daily, however there can be some delay before the person responsible for the vaccination register the vaccine in DVR.


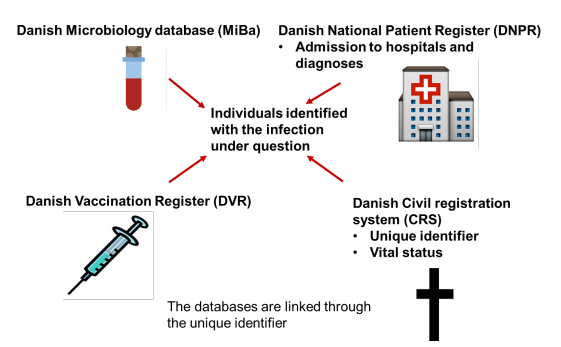


Figure S2. Databases or registries available from the State Serum Institute (SSI) of Denmark.

Table S1. List of ICD-CM diagnosis codes for pneumonia case definitions

|  | **ICD-9** | **ICD-10** |
| --- | --- | --- |
| Pneumonia | 489, 480.XX, 487.0, 485.XX-486.XX | J13.XX, J12.XX, J11.00, J18.XX |
| Pneumonia-related complications | 510.X, 511.0, 511.1, 511.89, 511.9, 513.X | J85, J86.0X, J86.9X, J90, J94.2X, R09.1X |

Table S2. List of ICD-CM diagnosis codes for underlying conditions definition

| **Underlying conditions** | **ICD-9** | **ICD-10** |
| --- | --- | --- |
| Diabetes mellitus (DM) | 249.XX, 250.XX | E08.XX - E13.XX |
| Cardivascular disease (CVD) | 401.XX-402.XX, 410.XX-414.XX, 427.3, 428.XX, 433.XX, 437.XX, 443.9, 740.XX-749.XX | I10.XX-I11.XX, I20.XX-I25.XX, I48.91, I50.XX, I63.XX, I65.XX, I67.XX, I73.9, Q20.X-Q28.X |
| Chronic lung disease (CLD) | 490-496.X | J44.0X, J44.1X, J43.XX-J45.XX |

Table S3. List of ICD-CM diagnosis codes for studied complications.

|  | **ICD-9** | **ICD-10** |
| --- | --- | --- |
| **Acute complications** | 510.X, 511.0, 511.1, 511.89, 511.9, 513.X | J85, J86.0X, J86.9X, J90, J94.2X, R09.1X |
| **Other complications** |  |  |
| Sepsis | 790.7, 995.91 | R78.81, A41.XX |
| Cerebrovascular events | 430.XX – 438.XX | I60.XX – I69.XX |
| Cardiovascular events | 410.XX – 414.XX, 420.XX – 427.XX | I20.XX-I25.XX, I30.XX-I52.XX |
| Acute Kidney Failure | 584.X | N17.XX |
| Respiratory failure/ARDS | 518.XX | J96.XX, J80 |

Table S4. Hospitalized PP incidence rates in Valencia, Spain, and Danish study populations, by site, sex, age group, underlying conditions, and year.

|  | **Number of (first) cases** | **Follow-up period** | **Incidence Rate**  **(95% CI)**  **[per 100,000 PY]** | **Number of (first) cases** | **Follow-up period** | **Incidence Rate**  **(95% CI)**  **[per 100,000 PY]** |
| --- | --- | --- | --- | --- | --- | --- |
|  | **Valencia, Spain** | | | **Denmark** | | |
| ***Overall*** | 8822 | 15,484,365 | 57(55.79 ,58.18) | 4897 | 19273277 | 25.41 (24.70; 26.13) |
| ***Sex*** |  |  |  |  |  |  |
| Male | 4970 | 7,124,656 | 69.8(67.83 ,71.72) | 2400 | 9223940 | \| 25.02 \| (24.99; \| 27.08) \| \| --- \| --- \| --- \| |
| Female | 3852 | 8,359,709 | 46.1(44.63 ,47.56) | 2497 | 10049337 | \| 24.85 \| (23.88; \| 25.84) \| \| --- \| --- \| --- \| |
| ***Age at entry*** |  |  |  |  |  |  |
| 50-55y | 423 | 3,089,201 | 13.7(12.42 ,15.06) | 305 | 3538457 | 8.629 (7.68; 9.64) |
| 55-60y | 556 | 2,679,701 | 20.7(19.06 ,22.55) | 460 | 3227115 | 14.25 (12.98; 15.62) |
| 60-65y | 658 | 2,353,374 | 28(25.86 ,30.18) | 597 | 3117937 | 19.15 (17.614; 20.75) |
| 65-70y | 898 | 2,137,355 | 42(39.31 ,44.85) | 701 | 3052955 | 22.96 (21.29; 24.73) |
| 70-75y | 1106 | 1,772,809 | 62.4(58.76 ,66.17) | 801 | 2452076 | 32.67 (30.44; 35.01) |
| 75-80y | 1275 | 1,436,771 | 88.7(83.94 ,93.75) | 646 | 1688480 | 38.26 (35.37; 41.33) |
| ≥80y | 3906 | 2,015,154 | 193.8(187.8 ,200.01) | 1387 | 2196257 | 63.15 (59.87; 66.57) |
| ***Underlying Condition*** | | | |  | | |
| Diabetes | 3111 | 2,836,882 | 109.7(105.84 ,113.59) | 545 | 993446 | 54.86 (50.35; 59.66) |
| CVD | 6684 | 7,746,889 | 86.3(84.22 ,88.37) | 1764 | 3585717 | 49.20 (46.93; 51.55) |
| CLD | 4189 | 2,359,921 | 177.5(172.17 ,182.96) | 731 | 497986 | 146.79 (136.34; 157.83) |
| ***Year*** |  |  |  |  |  |  |
| 2010 | 800 | 1,573,656 | 50.8(47.37 ,54.49) | 584 | 2022562 | 28.87 (26.58; 31.31) |
| 2011 | 921 | 1,607,783 | 57.3(53.64 ,61.11) | 577 | 2046423 | 28.20 (25.94; 30.59) |
| 2012 | 802 | 1,646,319 | 48.7(45.4 ,52.21) | 540 | 2078382 | 25.98 (23.84; 28.27) |
| 2013 | 867 | 1,678,606 | 51.7(48.27 ,55.21) | 520 | 2100328 | 24.76 (22.68; 26.98) |
| 2014 | 951 | 1,718,918 | 55.3(51.86 ,58.96) | 511 | 2134646 | 23.93 (21.91; 26.11) |
| 2015 | 1023 | 1,758,353 | 58.2(54.67 ,61.86) | 558 | 2169244 | 25.73 (23.63; 27.95) |
| 2016 | 964 | 1,802,542 | 53.5(50.16 ,56.97) | 529 | 2212335 | 23.91 (21.92; 26.04) |
| 2017 | 1111 | 1,830,180 | 60.7(57.19 ,64.38) | 516 | 2241384 | 23.02 (21.08; 25.10) |
| 2018 | 1383 | 1,868,009 | 74(70.19 ,78.04) | 562 | 2267973 | 24.78 (22.77; 26.92) |
| CI: Confidence interval; CLD: Chronic lung disease; CVD: Cardiovascular disease; VID: Valencia Health System Integrated Databases. Note: Sum of IPP and NIPP incident cases may differ from the overall sum of incident PP cases due to the people who had more than one IPP and/or NIPP incident case during the study period, as counts for PP cases were censored when first IPP or NIPP case appeared. | | | | | | |
